# Natural Products and Inflammation

**DOI:** 10.3390/molecules22010120

**Published:** 2017-01-12

**Authors:** Norbert Latruffe

**Affiliations:** Laboratoire BioPeroxIL, Faculté des Sciences Gabriel, Université de Bourgogne, 6, Blvd Gabriel, F-21000 Dijon, France; Norbert.Latruffe@u-bourgogne.fr; Tel.: +33-380-396-237

Inflammation (or inflammatory reaction) is the response to body aggression by a pathogen agent, an allergen, a toxic compound, a tissue lesion, etc. It can be a general phenomenon with fever and tiredness, or a local phenomenon with pain and edema. Inflammation is characterized by the production of various active signaling molecules, such as vaso-active amines (histamine/serotonin), prostaglandins, leukotriens, kininogens/kallikreins/kinins, complement factors, cytokines, and MMPs (Matrix MetalloProteinases)/TIMPs (Tissue Inhibitors of MetalloProteinases). All of the following pathologies present with a strong inflammatory component; infection, injury, vessel atherosclerosis, diabetes mellitus, obesity, cancer, osteo-arthritis, ocular diseases, demyelination, and brain pathologies associated with aging. Inflammation is a complex response that involves, among other interactions between activated lymphocytes, dendritic cells (i.e., antigen presenting cells or APCs) and monocytes, subsequently differentiated into macrophages. During this process, numerous cytokines are secreted by immune cells and by injured tissue non-immune cells as a consequence of cell-cell interactions.

The immunomodulation by various compounds, such as natural products, represents a promising preventive or therapeutic strategy against a number of pathological processes. Beside pro-inflammatory cytokines/interleukins, various lipid mediators produced through arachidonate metabolism also play a key role in inflammation-linked pathologies, such as atherosclerosis or cancers.

More than 27 original papers or review papers have reported the inhibitory effect of natural products on inflammation processes, at least in low grade inflammation. These compounds included plant polyphenols or derivatives: resveratrol [1], quercetin [2,3], edible plants (cactus [4], tomato [5]), herbal medicine [6] such as *Potentilla erecta* [7], coumarin derivatives [8], Euphorbia derivative Jolkinolide B [9], *Viscum album* [10], koumine, as alkaloid of *Gelsemium elegans* [11], soft medicine (*Anacardium* [12]), and different interesting compounds: the anti-inflammatory cosmetics (*Citrus bergamia* [13]), *Aspalathus linearis*, an African plant used as a drink [14], or Xanthone as a dye [15].

Besides reviews on anti-inflammatory properties of natural products [6,16], there are papers investigating natural compound-dependent anti-inflammatory mechanisms including the regulation of non-coding regulatory miRNAs [17], HDAC modulation [18], COX-2 sensitivity [19], Inflammasome targeting [20], and the use of coumarin derivatives [8]. In addition, this Special Issue reports papers related to the anti-inflammatory effect of natural products on different physiopathological processes: allergy [6], ocular system [1], keratinocytes [14], asthma [21], emphysema [22], respiratory tract [9], viral infection [23], immune cells [2,11,24,25], and the brain [4,15].

In conclusion, the large number and the diversity of papers in this proposed topic of Natural Products and Inflammation confirm the interest of this association. It contributed to reveal promising compounds and their potential interest in the immunmodulation.
